# Disrespect and abuse of women during childbirth in public health facilities in Arba Minch town, south Ethiopia – a cross-sectional study

**DOI:** 10.1371/journal.pone.0205545

**Published:** 2019-04-29

**Authors:** Gebresilasea Gendisha Ukke, Mekdes Kondale Gurara, Wanzahun Godana Boynito

**Affiliations:** 1 Department of Midwifery, College of Medicine and Health Sciences, Arba Minch University, Arba Minch, Ethiopia; 2 Department of Public Health, College of Medicine and Health Sciences, Arba Minch University, Arba Minch, Ethiopia; Greenebaum Cancer Center, Institute of Human Virology, University of Maryland School of Medicine, UNITED STATES

## Abstract

**Introduction:**

Disrespect and abuse of women during childbirth is one of the deterring factors to skilled childbirth utilization, especially in low and middle-income countries.

**Objective:**

The objective of this study was to assess the prevalence of women’s disrespect and abuse during childbirth in public health facilities in Arba Minch town, south Ethiopia.

**Methods:**

Institution-based cross-sectional study design was employed at all public health institutions in Arba Minch town, south Ethiopia. A systematic random sampling method was used to include 281 women who had given birth at public health institutions between January 01 and February 28, 2017. Data were collected through face to face interview by four data collectors and they were supervised by the principal investigator during the entire period of data collection. A semi-structured pretested questionnaire was used to collect the data. Epi info version 7.1.2.0 and SPSS version 24 were used to enter and analyze the data, respectively.

**Results:**

The overall prevalence of non-respectful care was 98.9%. The women’s right to information and informed consent was the most frequently violated right with a prevalence of 92.5% (95% CI: 90.9, 94.1) followed by non-dignified care (36.7, 95% CI: 34.9, 38.5), physical abuse (29.5%, 95% CI: 24.2, 34.8), discrimination (18.1%, 95% CI: 13.6, 22.6), non-confidential care (17.1%, 95% CI: 12.7, 21.5) and abandonment of care (4.3%, 95% CI: 3.1, 5.5). Rural residence, giving birth in the hospital, having no or low educational status and giving birth by cesarean route were factors significantly associated with specific women’s rights violations.

**Conclusions and recommendations:**

The prevalence of women’s disrespect and abuse during childbirth at the health care facilities in this study area is very high. Therefore, health managers need to work hard to tackle the problem.

## Introduction

Even though significant improvement of maternal health was observed in between 1990 and 2015, maternal mortality still continue to be a public health problem globally[1, 2]. In 2015, an estimated 303,000 women lost their lives due to easily preventable pregnancy and childbirth-related complications worldwide, 99% of which was contributed by low and middle-income countries[1].

As the majority of the maternal deaths occur during delivery and immediate postpartum periods, increasing skilled birth attendance has been one of the strategies proposed by experts to reduce maternal and neonatal morbidity and mortality[3–5]. The Safe Motherhood Initiative had therefore focused mainly on improving access to and utilization of skilled childbirth attendance and facility-based maternity care for the last several years[6, 7]. However, clients’ satisfaction with care provided which is an important element of quality care had not been given enough attention[8].

Even though Ethiopia is one of the countries commended for reducing maternal mortality, it is still among the countries with the highest maternal mortality ratio in the world (412 per 100, 000 live births)[9, 10]. To achieve the newly set sustainable development goal three (i.e. reducing the global maternal mortality ratio to less than 70 per 100,000 live births in 2030), Ethiopia needs tripling the former 2.3% annual maternal mortality reduction rate to 7.5%[1, 4].

Like that of maternal mortality, there is a big discrepancy in skilled birth attendance between high-income and low-income countries [11]. In Ethiopia, the proportion of women who are utilizing skilled birth attendance is increasing progressively from10% in 2011to 15% in 2014 and 26% in 2016 but it is still very low[9, 12, 13]. While there are many barriers to skilled birth utilization in low-income countries like low education [14–16], low economic status[16–18], rural residence and distance[14, 15]; sometimes problems related to care provider behavior and attitude are more important deterring factors than geographical and financial limitations to the utilization of skilled childbirth care[19, 20].

Most of the time, in low-income countries, women do not get their expected quality of care and level of respect by the health care providers at health care facilities[21, 22]. When the women are treated disrespectfully, their negative encounter with health workers during delivery leaves a long-lasting damage and emotional trauma. These concurrences adversely affect skilled birth attendance during their subsequent deliveries [7, 23, 24].

Respectful maternity care advocates suggest that safe motherhood must be expanded beyond the prevention of illness or death to include respect for women’s basic human rights including respect for women’s autonomy, dignity, feelings, choices, and preferences[7]. The Respectful Maternity Care Charter developed by the White Ribbon Alliance and respectful maternity care partners was based on a framework of human rights to avert the disrespect and abuse of childbearing women [7, 19, 25].

According to the charter, seven rights were drawn from the categories of disrespect and abuse which are not mutually exclusive: *Article 1*: Every woman has the right to be free from harm and ill-treatment. *Article 2*: Every woman has the right to information, informed consent and refusal, and respect for her choices and preferences, including the right to her choice of companionship during maternity care. *Article 3*: Every woman has the right to privacy and confidentiality. *Article 4*: Every woman has the right to be treated with dignity and respect. *Article 5*: Every woman has the right to equality, freedom from discrimination, and equitable care. *Article 6*: Every woman has the right to healthcare and to the highest attainable level. *Article 7*: Every woman has the right to liberty, autonomy, self-determination, and freedom from coercion[19].

Currently, respectful maternity care is a top priority in the World Health Organization (WHO) recommendations on intrapartum care for a positive childbirth experience. The WHO recommends provision of respectful maternity care in accordance with a human rights-based approach to decrease maternal morbidity and mortality and improve women’s experience of labor and childbirth and address health disparities[26].

However, disrespect and abuse during childbirth is common throughout the world[19]. It can occur at the level of contact between the client and the care provider, as well as through systemic failures at the health facility and health system level[19]. Various studies across different countries have shown a high prevalence and negative impact of disrespect and abuse in facility-based childbirth on skilled care utilization [19, 27–42].

Even if one recent study conducted in Kenya has shown a relatively lower (20%) prevalence of disrespect and abuse during childbirth, in Ethiopia, the few studies which have been conducted in the central and the northern part of the country have revealed a high prevalence of the problem[21, 22, 43]. According to one study conducted in Addis Ababa, the capital of Ethiopia in 2013, the overall prevalence of disrespect and abuse during childbirth was 78.6% [22]. Participants of another qualitative study which was carried out in the northern part of the country also described disrespect and abuse as the major obstacles to the utilization of institutional delivery services [43].

In the southern part of the country where more than 56 aboriginal ethnic groups with different culture, language and norms live, we could not find any published research regarding respectful maternity care. This study was, therefore, aimed at assessing the prevalence of disrespect and abuse of women during facility-based childbirth in Arba Minch town, south Ethiopia.

## Materials and methods

### Study area and period

This study was conducted in public health facilities at Arba Minch town. Arba Minch is the administrative town of Gamo Gofa zone, one of the 14 zones in Southern Ethiopian Nations, Nationalities and Peoples Region. The estimated population of Gamo Gofa zone for 2016/17 according to projection form 2007 Ethiopia Central Statistical Agency was 2,043,668[44]. Arba Minch town is located at a distance of 495 kilometers south of Addis Ababa. The estimated population of the town for the year 2016/17 is about 159,019[44].

There are two public health centers and one public hospital at Arba Minch town that are providing curative and preventive health services including maternity care to the community in and around the town. Arba Minch general hospital, the only hospital in the town has been serving as a referral hospital for patients from surrounding districts in the zone as well as from the surrounding zones. The institutional delivery coverage of Arba Minch town for the year 2016/17 was 84% [45]. The study was conducted in all the three health institutions: Arba Minch General Hospital, Shecha Health Center and Sikela health center. The study period was from January 1 –February 28, 2017.

### Study design

Institution-based cross-sectional study design was employed for this study. Women who utilized one of the three public health facilities for childbirth purpose except the ones who underwent elective cesarean section were included in the study.

### Sample size and sampling techniques

A single population proportion formula was used to estimate the sample. The expected level of disrespect and abuse of 79% which is obtained from one previous study conducted in Addis Ababa [22], 95% confidence, 5% precision, and 10% none-response rate were used for the calculation giving a sample size of 281. The average number of deliveries during the two months of data collection time at the three health institutions was 580 (400 at Arba Minch General Hospital, 120 at Sikela Health Center and 60 at Shecha health center). One hundred ninety four mothers from Arba Minch General Hospital, 58 from Sikela health center and 29 from Shecha health center were included in the study through proportionate sampling and every other woman was intervened.

### Study variables

#### Independent variables

**Socio-demographic characteristics:** Age, residence, income level, occupation, educational status.

**Obstetric characteristics:** Parity, history of antenatal care follow-up, history of previous institutional delivery, place of birth, type of delivery.

#### Dependent variable

*Non-respectful and abusive care*: Physical abuse, non-consented care, non-confidential care, non-dignified care, discrimination based on specific clients’ attributes, abandonment of care and detention in health facilities.

### Data collection and quality control

The data were collected by four midwife tutors (technical assistants) who are working in skill demonstration lab of the college of medicine and health sciences of Arba Minch University. The data collectors have been trained for two days on how to carry out their duty ahead of starting data collection. A pretest was carried out on 14 mothers prior to the actual study period to check the consistency of the questionnaire and the ability of the data collectors to carry out the duty. The questionnaire was modified based on the pretest results.

Exit survey was carried out during the discharge times from postnatal ward. Questions on socio-demographic and obstetric characteristics of the study participants and details of the seven respectful maternity care rights were collected through face to face interview. The principal investigator had been supervising the data collectors closely during the entire period of data collection. All the questionnaires were reviewed and checked for completeness every day by the investigators. Epi info version 7.1.2.0 was used for data entry to reduce possible errors during data entry. Each and every questionnaire was crosschecked with the entered data and all observed errors were corrected.

### Data processing and analysis

All the questionnaires were checked for completeness, coded and entered into Epi Info version 7.1.2.0 and then transported to SPSS version 24 software package for analysis. Descriptive statistics such as mean, percentage and standard deviation, were determined. Bi-variable logistic regression was done to determine the association between each independent variable and the outcome variables. Variables with a p-value less than 0.25 in bi-variable logistic regression were entered to multivariate logistic regression to adjust the effect of confounders on the outcome variables. Purposeful selection of covariates method was used for the final multivariable model. The degree of association between dependent and independent variables was determined using the odds ratio with a confidence interval of 95% and a p-value of 0.05[46].

### Ethical considerations

Different ethical procedures were attained for this study before the commencement. Primarily, ethical clearance was obtained from Ethical Review Committee of College of Medicine and Health Sciences, Arba Minch University. Secondly, an official letter of support was obtained from the College of Medicine and Health Sciences to Arba Minch General Hospital, Sikela and Shecha health centers. At the same time, written informed consent was obtained from each study participant after they were informed about the objective of the study and after they were assured confidentiality of the information they were giving. The participants were also informed that they have a full right to refuse participation in the study or to quit at any time during the interview.

## Results

### Socio-demographic characteristics of the study participants

A total of 281 women who had given birth at the public health institutions in Arba Minch town were interviewed and no woman approached refused to participate in the study. The mean age of the study participants was 28.5 years (SD ± 5.92). More than half of the women (57.3%) were housewives, 64.8% of them were urban residents, 98.6% were married and 65.1% of them have their own income. Almost half of the respondents were from Gamo ethnic group (48.4%) and Orthodox Christians (49.8%) **(Table 1).**

**Table 1:** Socio-demographic characteristics of the study participants (n = 281).

Variable	Frequency	Percent
**Age**		
< 20 years	12	4.3
21–34 years	214	76.2
≥ 35 years	55	19.6
**Religion**		
Orthodox	140	49.8
Protestant	112	39.9
Muslim	13	4.6
Others*	16	5.7
**Residence**		
Rural	99	35.2
Urban	182	64.8
**Ethnicity**		
Amhara	19	6.8
Gamo	136	48.4
Gofa	41	14.6
Konso/Derashe/Alle	13	4.6
Oromo	17	6.0
Wolyita	44	15.7
Others**	11	3.9
**Marital Status**		
Married	277	98.6
Divorced	1	0.4
Widowed	3	1.1
**Occupation**		
Daily laborer	15	5.3
House wife	161	57.3
Student	22	7.8
Private business	40	12.2
Governmental employee	43	15.3
**Income status**		
Have their own income	98	34.9
Don’t have their own income	183	65.1
**Educational status**		
Unable to read & write	56	19.9
Able to read & write	28	10.0
Elementary school (Grade 1–4)	51	18.1
Secondary school(Grade 5–8)	72	25.6
High /Preparatory school(Grade 9–12)	25	8.9
Above grade 12	49	17.4

* Traditional, Catholic and & Jehovah Witnesses

** Guragie, Tigrie & Kore

### Obstetric characteristics of the study participants

More than 95% of the study participants had history of ANC follow up during the recent pregnancy. Almost two-third of them had history of previous institutional delivery and 214(76.2%) had given birth via vaginal route. Kneeling was the preferred birthing position for 208 (74.0%) of the respondents **(Table 2).**

**Table 2:** Obstetrics characteristics of the study participants (n = 281).

Variable	Frequency	Percent
**Place of delivery**		
Arba Minch general hospital	194	69.0
Shecha health center	29	10.3
Sikela health center	58	20.7
**History of ANC follow up**		
Yes	268	95.4
No	13	4.6
**Parity**		
Primiparous	74	26.3
Multiparous	167	59.4
Grand multiparous	40	14.2
**History of previous institutional delivery**		
Yes	177	63.0
No	104	37.0
**Route/Type of delivery**		
Vaginal	214	76.2
Cesarean	67	23.8
**Time of delivery**		
Day	135	48.0
Night	146	52.0
**Number of birth attendants**		
1–2	121	56.5
3–4	74	34.6
5–8	19	8.9
**Sex of the main birth attendant**		
Female	153	71.5
Male	61	28.5
**Preferred birthing position**		
Kneeling	208	74.0
Laying on back /Lithotomy	67	23.8
Squatting	6	2.1
**Labor was augmented/induced**		
Yes	30	10.7
No	251	89.3
**Transfused with blood**		
Yes	1	0.4
No	280	99.6

### The overall prevalence of non-respectful and abusive care

While the seven women’s rights were not equally violated; ranging from 0% in the seventh right to more than 90% in the second right, overall 278 (98.9%) of the women have reported that they have faced at least one of the violations of the seven women’s rights. The next section describes violations of the individual right one by one.

#### Violation of article 1: Women’s right to be free from harm and ill-treatment (prevalence of physical abuse)

Two women (0.7%) have reported that the birth attendants have used physical forces (slapping) against them while they were in labor while 4 (1.4%) of the women reported that the birth attendants have threatened them with use of physical force. Among those women who gave birth vaginally, 64 had their perineum sutured because of perineal tear or after episiotomy. Among them 12 (18.8%) have reported that their perineum was sutured without the use of any anesthesia, 35 (16.4%) have reported that the birth attendants have pushed their abdomen down (used fundal pressure) to deliver their babies. One hundred thirty-six (63.6%) of the women who gave birth vaginally stated that they were not allowed to adopt the position of their choice to bear down. Regarding ambulation, 87 (31%) of the women were not allowed to ambulate before giving birth of which 26 (29.9%) were not told reasons for restricting ambulation while 21 (7.5%) of the women 18 of these (85.7%) finally underwent cesarean reported that they were restricted from any fluid during the course of labor. Overall, the prevalence of physical abuse was 29.5% (95% CI: 24.2, 34.8).

#### Factors associated with physical abuse

In bi-variable analysis: being a rural resident, having no ANC follow up during the recent pregnancy, having no previous history of institutional delivery, and giving birth at the hospital were factors associated with physical abuse. However, multivariable analysis demonstrated that being a rural resident and delivery in the hospital were the only factors which are significantly associated with physical abuse.

Women who gave birth at the hospital were about 2 times more likely to be physically abused compared with those who gave birth at the health centers (AOR = 2.82, 95% CI: 1.48, 5.35). Those women who were from rural areas were about 2 times more likely to face physical abuse than their urban counterparts (AOR = 1.85, 95% CI: 1.07, 3.18) (**Table 3**).

**Table 3:** Bivariable and multivariable analysis of factors associated with physical abuse during childbirth (n = 281).

Variables	Physical abuse		OR (95% CI)	
	Yes	No	COR (95% CI)	AOR (95% CI)
**Place of delivery**				
Hospital	68(35.1%)	126 (64.9%)	2.59(1.38,4.86)	**2.82 (1.48, 5.35)**
Health center	15(17.2%)	72(82.8%)	1	**1**
**Residence**				
Rural	36(36.4%)	63(63.6%)	1.64(0.96, 2.78)	**1.84(1.07, 3.18)**
Urban	47(25.8%)	135(74.2%)	1	**1**
**ANC follow up**				
No	6(46.1%)	7 (53.9%)	2.13 (0.69, 6.53)	*
Yes	77(28.7%)	191(71.3%)	1	
**History of previous institutional delivery**				
No	38(36.5%)	66(64.5%)	1.69 (1.00, 2.85)	*
Yes	45(25.4%)	132(74.6%)	1	

Hospital: p-value: 0.02, Rural residence: p-value = 0.027

* = Not significant in multivariate analysis

#### Violation of article 2: Women’s right to information, informed consent (prevalence of non-consented care)

The women’s right to information and informed consent was the most frequently violated right as reported by the study participants. Only 19 (6.8%) of the study participants have reported that the caregivers have introduce themselves during the admission time while about 208 (74%) reported that they were not told about the evaluation of their initial assessment by the caregivers. During the initial assessment, only 57(20.3%) of the respondents reported that they were encouraged to ask about unclear points. Among the 53 women for whom episiotomy was performed, only 21 (39.6%) had provided verbal consent. Out of the 67 women who underwent cesarean birth 61 (91%) reported that they were well informed before the procedure while 17 (56.7%) of the 30 women whose labor was augmented were told about the procedure in advance. Overall, 260 respondents reported that they were not consented at one point during their stay at the health institutions making the prevalence of non-consented care 92.5%, (95%CI: 90.9, 94.1).

#### Violation of article 3: Women’s right to privacy and confidentiality (prevalence of non-confidential care)

Women’s right to privacy and confidentiality is among the three women’s right which were less violated. Two hundred fifty-eight (91.8%) of the women responded that no one except those health care providers who were involved in their birth attendance has entered into the delivery room while they were naked. Almost all (97.2%) of the respondents have not heard the care providers sharing their secret information to others or they trust the care providers that they would not share their secret. The majority, 255 (90.7%) of the respondents were satisfied by the physical barriers or the use of curtains to keep their privacy during the course of labor and delivery. Overall, 233(82.9%) of the respondents reported that their care was confidential which makes the prevalence of non-confidential care 17.1% (95% CI: 12.7, 21.5).

#### Factors associated with non-confidential care

Rural residence and giving birth at the hospital were significantly associated with non-confidential care. Women who gave birth at the hospital were about 3 times more likely to report non-confidential care when compared to those who gave birth at the health centers (AOR = 3.34, 95% CI: 1.42, 7.87). Those women who were from the rural areas were about 2 times more likely to report non-confidential care when compared to their urban counterparts (AOR = 1.93, 95% CI: 1.01, 3.68) (**Table 4**).

**Table 4:** Bi-variable and multivariable analysis of factors associated with non-confidential during childbirth (n = 281).

Variables	Non-confidential care		OR (95% CI)	
	Yes	No	COR (95% CI)	AOR (95% CI)
**Place of delivery**				
Hospital	41(21.1%)	153 (78.9%)	3.06(1.31,7.14)	**3.34 (1.42, 7.87)**
Health center	7(8.0%)	80(92.0%)	1	**1**
**Residence**				
Rural	22(22.2%)	77(77.8%)	1.71(0.91, 3.22)	**1.93(1.01, 3.68)**
Urban	26(14.3%)	156(85.7%)	1	**1**

Hospital: p-value: 0.006, Rural residence: p-value = 0.046

#### Violation of article 4: Women’s right to be treated with dignity and respect (prevalence of non-dignified care)

Ninety-seven (34.5%) of the respondents reported that the health care providers were not talking to them politely while 8 (2.8%) of them reported that the care providers blame them for getting pregnant. About a quarter (23.1%) of the participants reported that the birth attendants have shouted at them to calm them down while they were in severe labor pain. More than one-third of the respondents (40.9%) responded that their relatives were not allowed to accompany them during the course of labor. Overall, 104 women reported at least one form of the violation of this right making the overall prevalence of non-dignified care to be 36.7% (95% CI: 34.9, 38.5).

#### Factors associated with non-dignified care

In the bi-variable analysis, low educational status or having no formal education, recent delivery by cesarean section and giving birth at the hospital were factors associated with non-dignified care. However, multivariate analysis has shown that births attended at the hospital and having no formal education or little schooling were the only factors which were significantly associated with non-dignified care.

Women who gave birth at the hospital were about 10 times more likely to face non-dignified care compared with those who gave birth at the health centers (AOR = 9.93, 95% CI: 4.37, 19.76). Those women who have no formal education were about 3 times more likely to be treated in a non-dignified way when compared to those who have at least completed secondary school (AOR = 3.17, 95% CI: 1.55, 6.49) (**Table 5**).

**Table 5:** Bi-variable and multivariable analysis of factors associated with non-dignified care during childbirth (n = 281).

Variables	Non-dignified care		OR (95% CI)	
	Yes	No	COR (95% CI)	AOR (95% CI)
**Place of delivery**				
Hospital	93(47.9%)	101 (52.1%)	7.09(3.463, 14.515)	**9.92 (4.37, 19.76)**
Health center	10(11.5%)	77(88.5%)	1	**1**
**Educational status**				
No formal education	27(48.2%)	29(51.8%)	2.02(1.08, 3.80)	**3.71(1.55, 6.49)**
Elementary school	30(38.0%)	49(62.0%)	1.33(0.75, 2.36)	**2.22(1.12, 4.04)**
Secondary or above	46(31.5%)	100(68.5%)	1	**1**
**Delivery type**				
Cesarean birth	33(49.3%)	34(50.7%)	1.20(1.14, 3.49)	0.78(042, 1.50)*
Vaginal delivery	70(32.7%)	144 (67.3%)	1	**1**

Hospital: p-value: 0.000, No formal educational: p-value = 0.002, Elementary school: p-value: 0.021

* = Not significant in multivariable analysis

#### Violation of article 5: Women’s right to equality, freedom from discrimination, and equitable care (prevalence of discrimination based on specific client attributes)

While no woman was discriminated because of her religion or because of her retroviral infection status, 2 (0.7%) women perceived that the birth attendants have discriminated them because of their traditional beliefs. Thirty-five (12.5%) of the respondents reported that they were discriminated (perceived) because they were from a rural area or because their educational status is low or have no formal education. Another 4 (1.4%) women have reported that they have discriminated because they were too young to give birth. Overall, 51 of the respondents felt discriminated because of their different attributes making the prevalence of discrimination 18.1% (95% CI: 13.6, 22.6).

#### Factors associated with discrimination

Educational status of the respondents, residence, place of delivery, type of delivery, history of previous health institutional delivery and occupation of the respondents were factors which were associated with perceived discrimination in bi-variable logistic regression analysis. In multivariable logistic regression analysis; place of delivery, residence, history of previous health institutional delivery and educational status of the respondents were factors which were significantly associated with perceived discrimination.

Perceived discrimination based on some women’s attributes was about 9 times more likely to be reported by women who gave birth at the hospital than at the health centers (AOR = 9.14, 95% CI: 3.17, 26.34). Those women who have no formal education were about 15 times more likely to have felt discriminated when compared to those who have at least completed secondary school (AOR = 14.82, 95% CI: 5.22, 39.25). Women who were from the rural areas were about 4 times more likely to have felt discriminated than those who are from urban area (AOR = 3.76, 95% CI: 1.70, 8.31). Those women who have no previous history of health institutional delivery were about 4 times more likely to have felt that they were discriminated than those who have a previous history of institutional birth (AOR = 4.44, 95% CI: 1.97, 9.10) (**Table 6**).

**Table 6:** Bivariable and multivariable analysis of factors associated with perceived discrimination (n = 281).

Variables	Perceived discrimination		OR (95% CI)	
	Yes	No	COR (95% CI)	AOR (95% CI)
**Place of delivery**				
Hospital	45(23.2%)	149 (76.8%)	4.08(1.67, 9.97)	**9.14(3.17, 26.34)**
Health center	6(6.9%)	81(93.1%)	1	**1**
**Educational status**				
No formal education	27(48.2%)	29(51.8%)	11.43(5.10, 25.63)	**14.82(5.22, 39.25)**
Elementary school	13(16.5%)	66(83.5%)	2.417(1.03,5.69)	**4.03(1.47, 11.09)**
Secondary or above	11(7.5%)	135(92.5%)	1	**1**
**Type of delivery**				
Cesarean birth	21(31.3%)	46(68.7%)	**2.80(1.47, 5.33)**	1.54(0.45, 5.28)***
Vaginal delivery	30(14.0%)	184(86.0%)	1	1
**Residence**				
Rural	35(35.4%)	64(64.6%)	5.67(2.94, 10.96)	**3.76(1.7, 8.313)**
Urban	16(8.8%)	166(91.2%)	1	**1**
**Previous history of health institutional delivery**				
No	32(30.8%)	72(69.2%)	3.70(1.96, 6.96)	**4.44(1.97,10.10)**
Yes	19(10.7%)	158(89.3%)	1	**1**
**Occupation**				
Occupations with no or low income*	47(23.7%)	151(76.3%)	6.15(2.14, 17.68)	1.54(0.45,5.28)***
Occupations with better income **	4(4.8%)	79(95.2%)	1	1

Hospital: p-value: 0.002, No formal educational: p-value = 0.000, Elementary school: p-value: 0.043, Rural residence: p-value = 0.000, History of health institutional delivery: p-value = 0.000

* = Housewives, students & daily laborers

** = Governmental employees and private business owners

*** = Not significant in multivariate analysis

#### Violation of article 6: Women’s right to healthcare and to the highest attainable level of health and continuous support (prevalence of abandonment of care)

Abandonment of care was the least to be reported by the study participants next to detention in health facilities. Only 12(4.3%) of the study participants responded that they were left alone for some period of time while they were in need of someone to be with them. However, none of the participants reported that she had given birth by herself in the health facility. The overall prevalence of abandonment of care is therefore 4.3%, (95% CI: 3.1, 5.5).

#### Violation of article 7: Women’s right to liberty, autonomy, self-determination, and freedom from coercion (prevalence of detention in health facilities)

From this study, no study participant responded that she or her families were detained in the health facilities for the issue of payment or damage to the health institutions’ equipment.

## Discussion

This study has investigated the status of disrespect and abuse of women during childbirth using the seven universal rights of childbearing women[7, 19]. Almost all (98.9%) of the women have reported at least one form of disrespect or abuse during their stay at the health care facilities.

This finding is similar with findings from one Nigerian study which was carried out in 2012 where 98% of the women had reported at least one form of disrespect[47]. However, it is higher than findings from a study conducted in Addis Ababa in Ethiopia in 2013 where the overall prevalence of disrespect and abuse was 76.8% and a Kenyan study conducted in 2011/12 where it was 20%[21, 22]. The higher prevalence in this study in comparison to the previous studies could be because of the differences in verification criteria. In our study, we have included the use of obsolete procedures which can put women’s health at risk like the use of fundal pressure to expel babies and suturing episiotomies or perineal tears without the use of local anesthesia as a physical abuse. It could also be explained by the differences in study settings as the previous Ethiopian study was conducted in Addis Ababa, the capital city of the country, where the social economic status of clients is expected to be higher. This very high prevalence of disrespect and abuse in most of the studies may indicate “normalization of disrespectful care” by clients as well as by health care providers in low-income countries[19, 42].

As the prevalence of violation of the seven women’s right ranged from zero (detention in health facilities) to nearly 100% (non-consented care), we have discussed the prevalence of each disrespect type separately as it will have different implications.

In this study, nearly one-third of the study participants have reported one form of physical abuse. This is similar with the findings from a study conducted in Nigeria in 2012 where the prevalence of physical abuse was 35.7% and the previous Ethiopian study where it was 32.9%[22, 47]. From this, we can learn that physical abuse has been persistently high since the last 5 years. However, as groups and criteria are not comparable no statements on evolution in the prevalence can be made.

Two mothers (Both from the hospital) have reported that the birth attendants have beaten them during the course of labor. This is similar to the results from a previous study conducted in 5 East and South African countries from 2009 to 2012 where two mothers reported the use of physical force like slapping against them[31] and another Ethiopian study conducted in Addis Ababa in 2013 where 4 mothers have reported that they have been beaten[22]. This shows that nearly1 in every 100 women who are in pain and stress of labor and need reassurance and pain-relief are attacked by health care providers which is inhuman.

The practice of fundal pressure in an attempt to assist spontaneous vaginal birth during the second stage of labor has been reported by significant proportion of women. This is similar with results from one previous study encompassed 5 countries in East & Southern Africa where Ethiopia was the only country where fundal pressure to aid delivery of babies was used [31]. This makes Ethiopia, one of the countries where some of the obsolete procedures are still practiced by some health care providers; although Cochrane Systematic Review which was released in March 2017 could not come up with a conclusion about the benefit or harm of the use of fundal pressure [48].

Not using local anesthesia for episiotomy or perineal tear repair in a place where there was no shortage of supply is also substantial. This is also reported by the study conducted in 5 countries where anesthesia for episiotomy or perineal tear repair was not used by care providers in Ethiopia[31]. Even though there is an improvement in the use of local anesthesia from not using at all in the previous study to more than 80% in this study, it should be used in all women with episiotomy or perineal tear. It is inhuman, unprofessional and also contributes to non-preference of health institution deliveries during future pregnancies[19].

Even though episiotomy by itself is not a physical abuse, routine episiotomies can result in unnecessary perineal scars which might lead to dyspareunia. However, no mother has reported that her legs were tied down during childbirth which indicates that some of the obsolete procedures have been abandoned as it was used to be practiced routinely [19].

Non-consented care is extremely high in this study as well as in a previous Ethiopian study [22]. It is more than 2-fold of the results from a study from Nigeria [47]. This may indicate that the right to information and informed consent is less considered as a clients’ right by Ethiopian health care providers when compared to other rights.

The proportion of proper informed consent increases with the potential risks or complications from the procedure (high for cesarean birth, moderate for augmentation and low for episiotomy). Performing procedures without clients’ consent was also reported by more than one-third of the respondents in the multi-country study and almost half of the participants in the previous Ethiopian study [22, 31]. This shows that the proportion of procedures without proper informed consent varies from setting to setting and also within the same setting according to the type of the procedure. This could be because of the nature of the procedure and variations in the health care providers.

The above interpretation can be supported by the results that very high proportion of women in this study as well as in the previous Ethiopian study reported that the health care providers have not introduced themselves at admission [22]. Less than one third of the women have been informed about findings of their initial assessment which will have high potential in preparing the clients on what to expect. This is relatively lower than the multi-country study finding where at least one-third have been informed [31]. If women do not take an active role in their healthcare decisions, they will not develop a better understanding of their choices and will less likely receive care consistent with their choices, values, and goals. [19]. This can be the main reason why the proportion of non-respectful care is very high in this study.

Regarding non-confidential care, a relatively small proportion of women responded that their privacy was not kept during the course of labor and delivery which is comparable with the results from the previous Ethiopian study where the prevalence was 21.4% [22]. However, it is lower than the finding from the previous multi-country study [Zanzibar (78%), Ethiopia (73%), Tanzania (46%), Kenya (35%), Madagascar (28%) and Rwanda (22%)[31]. It is also lower than the prevalence of non-consented care and physical abuse mentioned above in this study.

This could be because it is a factor which can be modified easily. For instance, physical barriers like curtains can be arranged by the administrators of the health institutions unlike that of human behavior which may take a longer period to change. It could also be because the number of health institutions participated in this study is very small and the presences of physical barriers like curtains mean non-or all for the health institution. However, the clients may report the inappropriate use of curtains during their course of labor.

More than one-third of the respondents have reported non-dignified care in this study which is relatively higher than the findings of other similar previous studies where it was between 10% to 20% [22, 31]. Some respondents perceived as if the care providers discriminated them because of their social-economic status which is also reported in other countries like Peru, Burkina Faso and Kenya as it was stated in Bowser and Hill landscape analysis[19].

The significantly higher proportion of women in reporting physical abuse, non-confidential care, non-dignified care and felt discriminated at hospital is also supported by the previous study conducted in Addis Ababa [22]. This could be attributed to workload and dissatisfaction by the hospital staffs, the presence of a different mix of health professionals with different backgrounds, and clients’ inability to communicate with the staffs due to language barriers (for those who are referred from rural health centers).

The high prevalence of non-confidential care at the hospital can be explained by the fact that hospital staffs are caring for a large number of clients at a time. When a client with life-threatening conditions like postpartum hemorrhage that needs team management is in a room, exposure of nearby clients is more likely.

The disproportionally high prevalence of physical abuse, non-confidential care and the feeling of being discriminated among those women who are from the rural areas is against their right as humans and will result in increased homebirths which are attended by unskilled persons in the future[19]. Obviously, this will pose a great challenge for the improvement of the maternal health of the country as more than 80% of the Ethiopian population resides in rural areas.

The higher prevalence of non-dignified care and the feeling of being discriminated among those women who have no formal education could be related to the violation of the right of the women to information. For instance, if a woman is told about the reasons why she should be alone in case of procedures which may not be comfortable for the relatives, the client might not feel this separation from her families as a violation of her right.

Even though the sixth article of the rights of childbearing women states that every woman has the right to healthcare and to the highest attainable level of health[7], women in labor are sometimes left alone. This result is relatively promising when compared to the previous studies where about 63.6% of the women who had given birth at hospitals in Addis Abba responded that they had been abounded during childbirth. But the figure is still high when compared to other similar studies in other African countries like Tanzania where it was 7.9%[22, 49]. This could be related to the number of health professionals per health institutions as the number is progressively increasing in Ethiopia, though the standard has not met yet.

The major strength of the study is the analysis of each violation of women’s rights separately. This indicates what to prioritize for intervention. Moreover, as the interview was carried out during discharge time, recall bias was minimized. However, the study would have been more comprehensive if it had been supported by direct observation and included more health institutions.

## Conclusions and recommendations

The prevalence of non-respectful maternity care at the health care facilities in the study area is very high. This needs health managers’ especial attention. Health care providers especially those who are working in the hospital need to be trained on respectful maternity care. The health care providers should also be oriented on the importance of the informed consent, on the need to avoid obsolete procedures and on appropriate management of episiotomies and perineal tears. Improving women education and empowering the rural women is needed to decrease the communication gaps between the health care providers and the women on what can result in disrespectful care during childbirth. Since this study was limited to urban center health facilities and made on the bases of interview and questionnaire, we recommend to other researchers to conduct further studies by involving rural health institutions and by including direct observation.

## Supporting information

S1 File Questionnaire_Amharic_version. (DOCX)

S2 File Questionnaire_English_version. (DOCX)

S3 File Operational definitions. (DOCX)

S1 Dataset (SAV)
