# Supplementary material for: Disrespect and abuse of women during childbirth in public health facilities in Arba Minch town, south Ethiopia – a cross-sectional study
Source: PLoS One. 2019 Apr 29;14(4):e0205545. doi: 10.1371/journal.pone.0205545 (PMC6488058; doi:10.1371/journal.pone.0205545)
Supplement: S1 File — (DOCX) [file pone.0205545.s001.docx]

**የፈቃድ ፎርም**

አርባ ምንጭ ዩኒቨርሲቲ የሕክምና እና ጤና ሳይንስ ኮሌጅ

እናቶች በአርባ ምንጭ ከተማ በሚገኙ የመንግስት ጤና ተቋማት በሚወልወዱበት ጊዜ የሚያገኙት የወሊድ አገልግሎት ምን ያህል አክብሮት የተሞላና ተገቢ ካልሆኑ አድራጎት ነጻ ስለሆመኑ ለማጥናት በአርባ ምንጭ ዩኒቨርሲቲ ህክምናና ጤና ሳይንስ ኮሌጅ የጥናት ቡድን የተዘጋጀ ቃለ-መጠይቅ፤ ጥቅምት 2009፤

ጤና ይስጥልኝ!_______________እባላለሁ፡፡የወሊድ አገልግሎት ምን ያህል በአክብሮት የተሞላና ተገቢ ካልሆኑ አድራጎት ነጻ ስለሆመሆኑ ለሚያጠኑ ለአርባ ምንጭ ዩኒቨርሲቲ ተመራማሪዎች መረጃ እየሰበሰብኩ ነኝ፡፡ የጥናቱ ዓላማ እናቶች በአርባ ምንጭ ከተማ በሚገኙ የመንግስት ጤና ተቋማት በሚወለወዱበት ጊዜ የሚያገኙት የወሊድ አገልግሎት ምን ያህል በአክብሮት የተሞላና ተገቢ ካልሆኑ አድራጎት ነጻ ስለሆመሆኑ ለማጥናት ነው፡፡ ለጥናቱ የሚረዱ መረጃዎችን ከአንቺ እንደማገኝ ተስፋ በማድረግ አንቺን የጥናቱ ተሳታፊ እንድተሆኚ መርጨሻለሁ፡፡ ከ 30 - 40 ደቂቃዎች ለሚወስድ ጊዜ ከአገልግሎት አሰጣጥ ጋር የተያያዙ ጥቂት ጥያቄዎች ልጠይቅሽ እፈልጋለሁ፡፡

በቃለ-መጠይቁ የመሳተፍና ያለመሳተፍ መብትሽ የተጠበቀ ነው፡፡ እየተሳተፍሽ ካልተመቸሽም በመሃል ማቋረጥ ትቸያለሽ፡፡ ጥያቄዎቼን ለመመልስ ጊዜ ካለሽና ፈቃደኛ ከሆንሽ ስምሽና ለሎች ስለአንቺ የሚገልፁ መገለጫዎች ስለማይመዘገቡ የሚትሰጪው መረጃ ሚስጥራዊነተ የተጠበቀ ይሆናል፡፡በቅንነት የሚትሰጫቸው መረጃዎች ግን ለወደፊቱ የወሊድ አገልግሎት ጥራትን በሻሻል በኩል ከፍተኛ ድርሻ ይኖረዋል፡፡ ዛሬ የሚትሰጪው መረጃ ወደፊት ከዚህ ጤና ተቋም ከምታገኚው አገልግሎት ጋር በፍጹም ተያያዥነት ስለሌው ምንም ስጋት ልገባሽ አይገባም፡፡ እናም በንቃት እና በታማኝነት ለመሳተፍ ፈቃደኛ ነሽ?  የምርምሩ ጥቅምና በምርምር ውስጥ የማከናውነውን ሚና ተረድቸ በጥናቱ ለመሳተፍ ተስማምቻለሁ.

*(አይደለም ከሆነ ማቆም፤ አዎን ከሆነ ያስፈርሙና እና ወደ መጠይቁ ይቀጥሉ::)*

አዎ አይደለም

የመረጃ ሰጪዋ ፊርማ ______________

የመረጃ ሰብሳቢዋ ፊርማ ______________

ቀን ____________________________

የተመራማሪዎቹ አድራሻ

1. መቅደስ ቆንዳሌ ኢሜል: kondale@gmail.com. ስልክ: 09 26 16 0903
2. ገ/ስላሴ ገንድሻ gebretecno@gmail.com ስልክ: 0934596503
3. ዋንዛሁን ጎዳና ኢሜል: [wanzanati2011@gmail.com](mailto:wanzanati2011@gmail.com) ስልክ 0913689198

**ስለፈቃድ ፎርም**ዝርዝር መረጃ

አርባ ምንጭ ዩኒቨርሲቲ የሕክምና እና ጤና ሳይንስ ኮሌጅ

እናቶች በአርባ ምንጭ ከተማ በሚገኙ የመንግስት ጤና ተቋማት በሚወልወዱበት ጊዜ የሚያገኙት የወሊድ አገልግሎት ምን ያህል አክብሮት የተሞላና ተገቢ ካልሆኑ አድራጎት ነጻ ስለሆመኑ ለማጥናት በአርባ ምንጭ ዩኒቨርሲቲ ህክምናና ጤና ሳይንስ ኮሌጅ የጥናት ቡድን የተዘጋጀ ቃለ-መጠይቅ፤ ጥቅምት 2009፤

ይህ የመረጃ ወረቀት እንድትሳተፊበት የምንጠይቀውን የጥናት ፕሮጀክት ለማብራራት የተዘጋጀ ነው፡፡

**የጥናቱ አርዐስት**

እናቶች በአርባ ምንጭ ከተማ በሚገኙ የመንግስት ጤና ተቋማት በሚወልወዱበት ጊዜ የሚያገኙት የወሊድ አገልግሎት ምን ያህል አክብሮት የተሞላና ተገቢ ካልሆኑ አድራጎት ነጻ ስለሆመኑ

**የዋና ተመራማሪ ስም፡-** መቅደስ ቆንዳሌ

**የድርጅቱ ስም፡-** አርባ ምንጭ ዩኒቨርሲቲ, የህክምና እና የጤና ሳይንስ ኮሌጅ

**የስፖንሰር አድራጊው ስም፡-** አርባ ምንጭ ዩኒቨርሲቲ

**የጥናቱ ፕሮጀክት ዓላማ**

የዚህ የምርምር ፕሮጀክት ዋና ዓላማ እናቶች በአርባ ምንጭ ከተማ በሚገኙ የመንግስት ጤና ተቋማት በሚወልወዱበት ጊዜ የሚያገኙት የወሊድ አገልግሎት ምን ያህል አክብሮት የተሞላና ተገቢ ካልሆኑ አድራጎት ነጻ ስለሆመኑ ለማጥናት ነው፡፡ እናቶች በመንግስት ጤና ተቋማት በሚወልወዱበት ጊዜ የሚያገኙት የወሊድ አገልግሎት ምን ያህል አክብሮት የተሞላና ተገቢ ካልሆኑ አድራጎት ነጻ ስለሆመኑ ማወቅ ለወደፊቱ የጤና ተቋማዊ የወሊድ ሽፋንን በማሳደግ በኩል ከፍተኛ ድርሻ ይኖረዋል፡፡ በመሆኑም ይህ ጥናት የእናቶች እና የጨቅላ ሕጻናት ህመም እና ሞት በመቀነስ በሚደረግ ርብርብ ወስጥ የራሱን አሻራ የሚያሳርፍ ይሆናል፡፡

**ሂደት**

ከአርባ ምንጭ ዩኒቨርስቲ ምርምሩን ለማካሄድ ፊቃድ አግኝተናል፡፡ወደ አርባ ምንጭ በሚገኙ የጤና ተቋማትም የትብብር ደብዳቤ ተጽፏል፡፡ጥናቱ በአርባ ምንጭ ከተማ በህዝብ ጤና ተቋማት የወሊድ አገልግሎት የሚያገኙ ሴቶች ያካትታል፡፡ ስለዚህ እርስዎ በዚህ ጥናት ለመሳተፍ ፍቃደኛ ከሆኑ እንዲሳተፉ ተመርጠዋል፡፡

ለመሳተፍ ፈቃደኛ ከሆኑ በጣም ደስ ይለናል፡፡ እናም የዚህን ጥናት አላማ በትክክል መረዳትዎን እና ስምምነትዎን ማየት እንፈልጋለን፡፡ በመጨረሻም ትክክለኛውን ምላሽዎን ብቻ እንዲሰጡ በአክብሮት እነንጠይቅዎታለን፡፡

**ጉዳት**

በዚህ ምላሽ ላይ በመሳተፍ ከምንወስድብዎት ጊዜ ውጭ (ለ 40 ደቂቃዎች) በምላሽ በመሳተፍ የሚያጋጥምዎት ምንም ዓይነት ጉዳት ወይም ምቾት ማጣት አይኖርም፡፡ በምዝገባ መጽሐፎዎች ውስጥ የተመዘገበ ማንኛውም የግል መረጃ አይወሰድም፡ ወደ ሌላ አካል አይዛወርም፡፡ እያንዳንዱ መረጃ በምስጢር ይቀመጣል፡፡

**ጥቅም**

በዚህ ጥናት መሳተፍሽ በወሊድ ጊዜ በእናቶች ላይ የሚደርሰውን ጫና ለማወቅ በጣም ይረዳናል፡፡ በጥናቱ ውጤት ላይ በመመርኮዝም ለወደፊት መፍትሔዎች ይዘጋጃሉ፡፡ ይሁን እንጂ በዚህ የምርምር ፕሮጀክት ላይ በመሳተፍሽ ምንም አይነት ጉዳት ወይም ቀጥተኛ ጥቅም አታገኝም፡፡

**ለመሳተፍ ማበረታቻዎች / ክፍያዎች**

በዚህ ፕሮጀክት ለመሳተፍ ማበረታቻ ወይም ክፍያ አይሰጥዎትም፡፡

**ሚስጢራዊነት**

ከርስዎ የተሰበሰበ መረጃ ስምሽ ሳይጠቀስ በምስጢር ይቀመጣል፡፡ በስምሽ ፋንታ በምስጢር ውስጥ ያለ የኮምፒተር ቁጥር ስለምሰጥ የእርስዎ ማንነት የሚገልጽ ነገር አይኖርም፡፡

**የመተው ወይም የማቋረጥ መብት**

በዚህ ምርምር ውስጥ ላለመሳተፍ ሙሉ መብት አለዎት፡፡ ከፈለጉ መጠይቁን ከጀመሩ በኋላ መሀል ላይ ማቋረጥም ይችላሉ፡፡

የተመራማሪዎቹ አድራሻ

ይህ የምርምር ፕሮጀክት በአርባ ምንጭ ዩኒቨርስቲ የምርምር ስነ-ምግባር ኮሚቴ ፀድቋል፡፡ ማንኛውም ጥያቄ ቢኖርዎት የሚከተለውን ግለሰብ ማነጋገር ይችላሉ ፤ እንዲሁም በማንኛውም ጊዜ መጠየቅ ይችላሉ.

1. መቅደስ ቆንዳሌ ኢሜል: kondale@gmail.com. ስልክ: 09 26 16 0903
2. ገ/ስላሴ ገንድሻ gebretecno@gmail.com ስልክ: 0934596503
3. ዋንዛሁን ጎዳና ኢሜል: [wanzanati2011@gmail.com](mailto:wanzanati2011@gmail.com) ስልክ 0913689198

**ክፍል I. ስለ ጤና ተቋሙ አጠቃላይ መረጃ**

| **ተ.ቁ** | | **ጥያቄ** | **መልስ** | **ምርመራ** |
| --- | --- | --- | --- | --- |
|  | የጤና ተቋሙ ስም | | 1. አ/ምንጭ አጠቃላይ ሆስፒታል 2. ሼቻ ጤና ጣቢያ 3. ሲቀላ ጤና ጣቢያ |  |

**ክፍል II ማህበራዊ ኩነቶች**

| **ተ.ቁ** | **ጥያቄ** | **መልስ** | **ምርመራ** |
| --- | --- | --- | --- |
|  | ዕድሜዎ ስንት ነው? | _______________ ዓመት |  |
|  | ሥራዎ ምንድነው? | 1. የቤት እመቤት 2. የመንግስት ሠራተኛ 3. የግል ሥራ/ንግድ/ 4. ሌላ(ይገለፅ) |  |
|  | የየትኛው ብሔር ተወላጅ ነዎት? | 1. ጋሞ 2. ጎፋ 3. ወላይታ 4. አማራ 5. ኦሮሞ 6. ሌላ(ይገለፅ) |  |
|  | የየትኛው ሀይማኖት ተከታይ ነዎት? | 1. ኦርቶዶክስ 2. ፕሮቴስታንት 3. ሙስልም 4. ሌላ(ይገለፅ______________ |  |
|  | የትዳር ሁኔታዎ? | 1. ያገባች 2. የተፋታች 3. ላገበች 4. ባል የሞተባት 5. የተለያዩ |  |
|  | የትምህርት ደረጃወዎስ? | 1. ያልተማረች 2. መፃፍና ማንበብ የሚትችል 3. አንደኛ ደረጃ (ከ1ኛ -4ኛ ክፍል) 4. ሁለተኛ ደረጃ(ከ5ኛ-8ኛ ክፍል) 5. ከፍተኛ/መሰናዶ.(ከ9ኛ -12ኛ ክፍል) 6. ከ12ኛ ክፍል በላይ |  |
|  | የትዳር ጓደኛዎ የትምህርት ደረጃስ? | 1. ያልተማረ 2. መፃፍና ማንበብ የሚችል 3. አንደኛ ደረጃ (ከ1ኛ -4ኛ ክፍል) 4. ሁለተኛ ደረጃ(ከ5ኛ-8ኛ ክፍል) 5. ከፍተኛ/መሰናዶ.(ከ9ኛ -12ኛ ክፍል) 6. ከ12ኛ ክፍል በላይ |  |
|  | የወር ገቢሽ ምን ያህል ብር ይሆናል? | ___________ ብር | ከለሌ 0 ይባል |
|  | የባለቤትዎ የወር ገቢ ምን ያህል ብር ይሆናል? | ___________ ብር | ባል NA ከለሌ ይበሉ |
|  | የት ነው የሚኖሩት | 1. ከተማ 2. ገጠር |  |

**ክፍል III የሥነ-ወሊድ ታርክ**

| **ተ.ቁ** | **ጥያቄ** | **መልስ** | **ምርመራ** |
| --- | --- | --- | --- |
|  | በአጠቃላይ ስንት ጊዜ ነው የወለዱት? | _____________ ጊዜ |  |
|  | ለመጨረሻው እረግዝና የቅድሜ ወሊድ ክትትል አድርገው ነበር? | 1. አዎን 2. አይደለም |  |
|  | ከዚህ በፊት በመንግስት የጤና ተቋም ወልደው ያውቃሉ? | 1. አዎን 2. አይደለም |  |
|  | በአሁኑ ወሊድ ጊዜ ስንት ሰው ንው ያዋለደሽ/ያገዘዥ? | _______________ |  |
|  | በማዋለዱ ሂደት ውስጥ በዋናነት ያዋለደው ማን ነው?(ልጁን ይዞ ያወጣው) | 1. ሚድዋይፍ 2. ነርስ 3. ዶክተር 4. እንተርን ዶክተር 5. ተማሪ 6. የጤና መኮንን 7. አይ. ኢ.ኤስ. ኦ 8. አላውቅም | የደምበኛዋን ቻርት ይመልከቱ |
|  | በዋናነት ያዋለደሽ ሰው ወንድ ነው ሴት ነች? | 1. ሴት 2. ወንድ |  |
|  | ቀን ነው ወይስ በማታ ላይ ነው የወለድሸው? | 1. ቀን 2. ማታ |  |

**ክፍል IV አክብሮት የተሞላው የወሊድ ገልግሎትን በተመለከተ**

| **1** | **አካላዊ ጥቃት** | | |
| --- | --- | --- | --- |
| **ተ.ቁ** | **ጥያቄ** | **መልስ** | **ምርመራ** |
|  | በምጥ ህመም ላይ በነበርሽ ጊዜ ያዋለዱሽ ባለሙያዎች በአንቺ ላይ አካላዊ ጥቃት አድርሰው ነበር? ማለትም **የመምታት፡ የመቆንጠጥ፡ በጥፊ የመምታት** የመሳሰሉ ተግባራትን ፈፅመውብሽ ነበር? | 1. አዎን 2. አይደለም | አዎን ከሆነ ጠቆር ካሉትውስጠ ያክብቡ |
|  | በምጥ ህመም ላይ በነበርሽ ጊዜ ያዋለዱሽ ባለሙያዎች በአንቺ ላይ አካላዊ ጥቃት ባያደርሱም፡ ዝም ካላልሽ ወይም እነሱ ያሉትን ትእዛዘዝ ካለፈፀምሽ እመታሸላሁ ብለው ያስፈራራሽ ሰው ነበር? | 1. አዎን 2. አይደለም |  |
|  | ለመውለድ ወደ መውለጃ አልጋ ላይ በወጣሽ ጊዜ እግርሽ በገመድ ታስሮ ነበር? | 1. አዎን 2. አይደለም |  |
|  | ከወለድሽ በኃላ ስፌት ተደርጎልሽ ነበር ? | 1. አዎን 2. አይደለም | አይደለም ከሆ ወደ 6 ይህዱ |
|  | ከሆነ ማደንዘዣ ተሰጥቶሽ ነበር ወይስ እያመመሽ ነው የተሰፋሽው? | 1. በማደነንዘዣ 2. ያለ ማደንዘዣ |  |
|  | አንቺ ለማማጥ/ለመውለድ የሚትመርጭው እንደት ነው? | 1. በእምብርክክ 2. ቁጥጥ በማለት 3. በጀርባ በመተኛት 4. ሌላ___ |  |
|  | በአሁኑ ምጥና ወሊድ ጊዜ ያገላገሉሽ ባለሙያዎች በምትፈልጊው ቦታ/አቀማመጥ ሆነሽ እንድታምጪ ፈቅደውልሽ ነበር ? | 1. አይደለም 2. አዎን |  |
|  | በምጥሽ ጊዜ ከቦታ ቦታ እንድተቀሳቀሽ ተፈቅዶልሽ ነበር ? | 1. አይደለም 2. አዎን | አዎን ከሆ ወደ 10 ይህዱ |
|  | ካለተፈቀደልሽ ምክንያቱ ተነግሮሽ ነበር? ለምሳሌ ምጥሽ ስለፋፋመ ወይም በሌላ ህመም ምክንያት ወዘተ… | 1. አይደለም 2. አዎን |  |
|  | ያገላገሉሽ ባለሙያዎች ልጅሽን በሚያወጡበት ጊዜ ሆድሽን ከላይ ወደ ታች ገፍተው ነበር (used fundal pressure)? | 1. አዎን 2. አይደለም |  |
|  | በምጥሽ ጊዜ ምንም ዓይነት ፈሳሽ ነገር እንዳትጠጭ ተደርገሽ ነበር ? | 1. አዎን 2. አይደለም |  |
|  | ያገላገሉሽ ባለሙያዎች ቤተሰቦችሽን የወለድሽበትን አልጋ ወይንም ክፍሉን እንድያፀዱ አስገድደው ነበር ? | 1. አዎን 2. አይደለም |  |

| **2** | **በሙሉ ፈቃደኝነት ላይ ያልተመሠረተ ክብካቤ** | | |
| --- | --- | --- | --- |
| **ተ.ቁ** | **ጥያቄ** | **መልስ** | **ምርመራ** |
|  | ያገላገሉሽ ባለሙያዎች ወደ ጤና ተቋሙ በምትመጡበት ጊዜ ለአንቺንና ለቤተሰቦችሽ ራሳቸውን አስተዋውቀው ነበር? | 1. አይደለም 2. አዎን |  |
|  | ባለሙያዎቹ መጀመሪያ ላይ እንችን በመረመሩ ጊዜ የምርመራውን ውጤት ማለትም የምጥሽ ደረጃና ሌሎች ሁኔታዎችን ነግረው ነበር? | 1. አይደለም 2. አዎን |  |
|  | ባለሙያዎቹ በምርመራው ጊዜ ስላልገቡሽ ነገሮች ጥያቄ እንድትጠይቅ አበረታትተውሽ ነበር ? | 1. አይደለም 2. አዎን |  |
|  | ባለሙያዎቹ ወደ ተቋሙ ከገባሽበት ጊዜ ጀምሮ ስለምጥሸ እየሆኔ ያለወን ነገር ከሥር ከሥር እየነገሩሽ ነበር ? | 1. አይደለም 2. አዎን |  |
|  | ልጁ በምወጣበት ጊዜ ለማሕጸን ጥበት ተብሎ ተቆርጠሸ ነበር? | 1. አዎን 2. አይደለም | አይደለም ከሆ ወደ 7 ይህዱ |
|  | ከተቆረጥሽ ለምን አንደሚትቆረጪ አስረድተወሽ እና ከመቁረጣቸው በፊት የአንችን ፈቃደኝነት ጠይቀው ነበር ? | 1. አይደለም 2. አዎን |  |
|  | የወለድሽው በሆድ በኩል በተደረገ ቀዶ ጥገና ነበር ? | 1. አዎን 2. አይደለም | አይደለም ከሆ ወደ 9 ይህዱ |
|  | ከሆነ ለምን አንደሆነ አስረድተወሽ እና ከኦፕራሲዮኑ በፊት የአንችን ፈቃደኝነት ጠይቀው እና መስማማትሽ አስፈርመውሽ ነበር ? | 1. አይደለም 2. አዎን |  |
|  | በምጥሽ ጊዜ የማማጫ መድሃኒት ተሰጥቶሽ ነበር ? | 1. አዎን 2. አይደለም 3. አላወቅሁም | አይደለም ከሆ ወደ 11 ይህዱ |
|  | ተሰጥቶሽ ከሆነ ለምን አንደምሰጡሽ አስረድተወሽ እና ከመስጠታቸው በፊት የአንችን ፈቃደኝነት ጠይቀው ነበር ? | 1. አይደለም 2. አዎን |  |
|  | በቆይታሽ ደም ተሰጥቶሽ ነበር ? | 1. አዎን 2. አይደለም | አይደለም ከሆ ወደ 13 ይህዱ |
|  | ተሰጥቶሽ ከሆነ ለምን አንደምሰጡሽ አስረድተወሽ እና ከመስጠታቸው በፊት የአንችን/የበተሰብሽን ፈቃደኝነት ጠይቀው ነበር ? | 1. አይደለም 2. አዎን |  |
|  | ባለሙያዎቹ በቀዶ ጥገና እድትወልጅ የማስገደድ ሁኔታ አሳይተው ነበር ? | 1. አዎን 2. አይደለም |  |

|  | **ምስጥራዊነቱ ያልተጠበቀ ክብካቤ** | | |
| --- | --- | --- | --- |
| **ተ.ቁ** | **ጥያቄ** | **መልስ** | **ምርመራ** |
|  | በምጥና በወሊድሽ ጊዜ ሌሎች ሰዎች እንዳያዩሽ ተብሎ መጋረጃ ነገር ተጠቅመው ነበር ? | 1. አይደለም 2. አዎን |  |
|  | ራቁተሽን በመውለጃ አልጋ ላይ በነበርሽ ጊዜ ከባለሙያዎቹ በቀር ሌላ ሰው ወደ ማዋለጃ ክፍሉ ገብቶ አይቶሸ ነበር? | 1. አዎን 2. አይደለም |  |
|  | ያዋለዱሽ ባለሙያዎች ምስጥርሽን ለሌሎች ለማይመለከታቸው ሰዎች አካፍለው ነበር? | 1. አዎን 2. አይደለም 3. አላወቅሁም |  |

|  | **አክብሮት የሌለው ክብካቤ** | | |
| --- | --- | --- | --- |
| **ተ.ቁ** | **ጥያቄ** | **መልስ** | **ምርመራ** |
|  | በምጥሽና በመውለጃሽ ጊዜ ሁሉ ባለሙያዎቹ ትህትና በሞላበት አነጋገር ነበር ካንቺ ጋር ስነጋገሩ የነበረው? | 1. አይደለም 2. አዎን |  |
|  | ባለሙያዎቹ አሳፋሪ ነው የምትይ ድርጊት ፈፅመውብሽ ነበር? | 1. አዎን 2. አይደለም |  |
|  | ባለሙያዎቹ የምጡ ህመም/ስቃይ ስበዛብሽና መቋቋም አቅቶሽ ስትጮህ መጀመሪያ ለምን አረገዝሽ፤ለእኔ ነው እንዴ ያረገዝሽው፡ የህን ስታውቅ ለምን አረገዝሽ… ምናምን በማለት አንቺን ኮንነው ነበር? | 1. አዎን 2. አይደለም |  |
|  | ባለሙያዎቹ ስቃዩ በዝቶብሽ ስትጮህ፡ ዝም በይ! በማለት ተቆጥተቅሽ ነበር? | 1. አዎን 2. አይደለም |  |
|  | በላሙያዎቹ ካንቺ ጋር የመጡ ቤተሰቦችሽ/ባለቤትሽ/ካንቺ ጋር እንድሆኑ የፈለግሻቸውን ሰዎች ወደ ማማጫ ክፍል እንድገቡ ፈቅደው ነበር ? | 1. አይደለም 2. አዎን |  |

|  | **አድልዎ ያለበት ክብካቤ** | | |
| --- | --- | --- | --- |
| **ተ.ቁ** | **ጥያቄ** | **መልስ** | **ምርመራ** |
|  | ባለሙያዎቹ በባህልሽ ምክንያት አድልኦ አድርሰውብሽ ነበር? | 1. አዎን 2. አይደለም 3. NA |  |
|  | ባለሙያዎቹ በሀይማኖትሽ ምክንያት አድልኦ አድርሰውብሽ ነበር? | 1. አዎን 2. አይደለም |  |
|  | ባለሙያዎቹ በትምህርት ደረጃሽ ዝቅተኛ በመሆኑ ምክንያት አድልኦ አድርሰውብሽ ነበር? | 1. አዎን 2. አይደለም 3. NA |  |
|  | ባለሙያዎቹ የገጠር ሰው በመሆንሽ/ከሩቅ አከባቢ ስለመጣሽ/ አድልዎ አድርሰውብሽ ነበር? | 1. አዎን 2. አይደለም 3. NA |  |
|  | ባለሙያዎቹ ኤች. አይ. ቪ. በደምሽ ውስጥ ስለምገኝ አድልኦ አድርሰውብሽ ነበር? | 1. አዎን 2. አይደለም 3. NA |  |
|  | ባለሙያዎቹ ዕድሜሽ ትንሽ/በጣም የገፋ በመሆኑ አድልኦ አድርሰውብሽ ነበር? | 1. አዎን 2. አይደለም 3. NA Yes 4. No |  |

|  | **ቀጣይነት ያለው የቅርብ ክትትል የጎደለው ክብካቤ** | | |
| --- | --- | --- | --- |
| **ተ.ቁ** | **ጥያቄ** | **መልስ** | **ምርመራ** |
|  | በምጥሽ ወቅት የጤና ባለሙያዎቹን እርዳታ በሚያስፈልግሽ ጊዜ ለብቻሽ ያለ ባለሙያዎች ክፍሉ ውስጥ የቆየሽበት አጋጣም ነበር? | 1. አዎን 2. አይደለም |  |
|  | ባለሙያዎች ካንቺ አጠገብ ባለመኖራቸው ልጅሽን የወለድሽው ለብቸቻሽ/በራስሽ ነበር? | 1. አዎን 2. አይደለም |  |
|  | በምጥሽ ወቅት ለህይወትሽ አስጊ የሆነ ነገር ተከስቶ የድረሱለኝ ጥሪ አሰምተሸ ሰሚ የጣሽበት አጋጣሚ ነበር? | 1. አዎን 2. አይደለም |  |

|  | **አላግባብ በጤና ተቋማት እናቶችን ማቆየት** | | |
| --- | --- | --- | --- |
| **ተ.ቁ** | **ጥያቄ** | **መልስ** | **ምርመራ** |
|  | በክፍያ ወይንም የጤና ተቋሙን ንብረት ላይ ጉዳት አደረሳችሁ በምል ምክንያት ካለወትሮው በጤና ተቋሙ እንድትቆይ ተደርገሽ ነበር? | 1. አዎን 2. አይደለም |  |

ስለትብብርዎ እናመሰግናለን!
