# Supplementary material for: Disrespect and abuse of women during childbirth in public health facilities in Arba Minch town, south Ethiopia – a cross-sectional study
Source: PLoS One. 2019 Apr 29;14(4):e0205545. doi: 10.1371/journal.pone.0205545 (PMC6488058; doi:10.1371/journal.pone.0205545)
Supplement: S2 File — (DOCX) [file pone.0205545.s002.docx]

## CONSENT FORM

## Arba Minch University College of Medicine and Health Sciences

## Consent form for a research project entitled disrespect and abuse of women during childbirth in public health facilities in Arba Minch town

**Hello**!

My name is _______________. I am working for investigators from Arba Minch University who are doing a research on the status of disrespectful maternity care in Arba Minch town. I have identified you as a study participant hoping that you would be willing to help me by providing some information. I would like to ask you a few questions about your sociodemographic characteristics and your history related to delivery service utilization which may take 30 – 40 minutes.

The goal of this study is to assess the status of disrespectful and abusive maternity care during facility-based childbirth in public health facilities in Arba Minch town. All information you provide will be kept confidential. I will not include any identifiers, such as your name or exact address. Your role in the success of the research is important and I appreciate your contribution to the research.

You have a full right to refuse part or the whole questionnaires & no one enforces you to do so. However, your honest participation and answers to the questionnaire will help us in a better understanding of the problem and give guidance on how to intervene in the study area. So are you willing to participate actively and honestly?

I understood about the advantage of the research, the roles I will have in the research and have agreed to participate in the research. (*If yes, let her sign and go ahead, if No stop here*.)

Yes Signature of the participant _____________ No

Signature of the data collector __________________________

Date: ______________________________________________

Contact persons:

1. Mekdes Kondale Email: [kondale@gmail.com](mailto:kondale@gmail.com). Phone: 09 26 16 0903
2. Gebresilasea Gendisha Email: [gebretecno@gmail.com](mailto:gebretecno@gmail.com) Phone: 0934596503
3. Wanzahun Godana Email: [wanzanati2011@gmail.com](mailto:wanzanati2011@gmail.com) Phone: 0913689198

## INFORMATION SHEET

## Arba Minch University College of Medicine and Health Sciences

## Information Sheet for a research project entitled disrespect and abuse of women during childbirth in public health facilities in Arba Minch town

This information sheet is prepared to explain the research project that you are asked to join by a group of research investigators.

**Title of the Research Project**

Disrespect and abuse of women during childbirth in public health facilities in Arba Minch town, South Ethiopia

Name of the Principal Investigator: Mekdes Kondale

**Name of the organization:** Arba Minch University, College of Medicine and Health Sciences

**Name of the sponsor:** Arba Minch University

**Purpose of the research project**

The main aim of this research project is to assess the status of disrespect and abuse of women during childbirth in public health facilities in Arba Minch town, South Ethiopia.

Assessing the status of non-respectful and abusive maternity care during facility-based is very important to increase institutional delivery coverage which in turn decreases maternal and neonatal morbidity and mortality as the results of this study will be used to design appropriate intervention programs to address the problem in the study area.

**Procedure**

Permission was processed from Arba Minch University to administrators of the respective health facilities in Arba Minch town. The study involves women who utilize public health facilities in Arba Minch town for delivery services. You are selected to be one of the study participants if you are willing to take part in this study and we kindly invite you to take part in our project.

If you are willing to participate, we are so happy and we need you to clearly understand the aim of this study and show your agreement. Finally, you are kindly requested to give your genuine response.

**Risk and/or discomfort**:

There is no any risk or discomfort that you will face by participating in this research except dedication of time (a maximum of 40 minutes) for responding. Any personal information registered in registration books will not be copied and transferred to other bodies. Every piece of information will be kept confidentially.

**Benefits**

Your participation is definitely important to know the level of disrespect and abusive care during facility-based childbirth and to design an appropriate strategy to maternity care services in this area. The findings of this study will be used to implement the intervention and reveal out the problem related to respectful maternity care. There is no risk or direct benefit in participating in this research project.

**Incentives/payments for participating**

You will not be provided with any incentives or payment to take part in this project.

**Confidentiality**

The information collected from you will be kept confidential and stored in a file, without your name by assigning a code number to it. And hence no report of the study ever identifies you.

**Right to refuse or withdraw**

You have a full right to refuse from participating in this research. You have also a full right to withdraw from this study at any time you wish.

**Person to contact**

This research project was reviewed and approved by the ethical committee of Arba Minch University. If you have any question you can contact the following individual and you may ask at any time you want.

Name: Mekdes Kondale Tel: 09 26 16 09 03 E-mail: [kondale@gmail.com](mailto:kondale@gmail.com) Or Gebresilasea Gendisha [gebretecno@gmail.com](mailto:gebretecno@gmail.com) Phone 09 34 59 65 03 Or Wanzahun Godana Email: [wanzanati2011@gmail.com](mailto:wanzanati2011@gmail.com) Phone 0913689198

## QUESTIONNAIRE (ENGLISH VERSION)

**Arba Minch University College of Medicine and Health Sciences**

Questionnaire to assess the status of disrespect and abuse of women during childbirth in public health facilities in Arba Minch town, South Ethiopia

**Part I. General Information about the health institutions**

| **Instruction: Circle the number(s) under the answer section according to the respondents’ answer or write the response on the blank space provided. NB: Do not forget skipping when appropriate.** | | | |
| --- | --- | --- | --- |
| **SN** | **Question** | **Answer** | **Remarks** |
|  | Name of the health institution | 1. Arbaminch General Hospital 2. Shecha Health Center 3. Sikela Health Center |  |

**Part-II Socio-demographic characteristics**

| **SN** | **Question** | **Response** | **Remarks** |
| --- | --- | --- | --- |
|  | How old are you? | _______________ years |  |
|  | What is your occupation? | 1. House wife 2. Government employee 3. Private business 4. Other (Specify) ______________ |  |
|  | To which ethnic group do you belong? | 1. Gamo 2. Gofa 3. Wolayta 4. Amhara 5. Oromo 6. Other (Specify) ____________ |  |
|  | To which religious groups do you belong? | 1. Orthodox (Christian) 2. Protestant (Chrsitian) 3. Muslim 4. Other (Specify) ______________ |  |
|  | What is your marital status? | 1. Married 2. Divorced 3. Single 4. Widowed 5. Separated |  |
|  | What is the highest grade you completed? | 1. No formal education /can’t read & write 2. No formal education but can read & write 3. Elementary school (grade 1 -4) 4. Secondary school(Grade 5-8) 5. High school/prep.(grade 9 -12) 6. Above grade 12 |  |
|  | What is the educational level of your husband? | 1. No formal education /can’t read & write 2. No formal education but can read & write 3. Elementary school (grade 1 -4) 4. Secondary school(Grade 5-8) 5. High school/prep.(grade 9 -12) 6. Above grade 12 |  |
|  | What is your monthly income level in Ethiopian Birr (ETB)? | ___________ ETB | Write 0 if the respondent does not has her own income |
|  | What is the monthly income level of your husband? | ___________ ETB | Write NA if no husband |
|  | Where are you living now? (Residence) | 1. Urban area 2. Rural area |  |

**Part III Obstetric characteristics**

| **SN** | **Question** | **Response** | **Remarks** |
| --- | --- | --- | --- |
|  | How many times did you give birth? (Parity) | _____________ times |  |
|  | Do you have ANC follow up during the current pregnancy? | 1. Yes 2. No |  |
|  | Do you have a history of previous institutional birth | 1. Yes 2. 2. No |  |
|  | How many birth attendants did assist you during the current birth? | _______________ |  |
|  | Who was the main birth attendant during this birth (the person who deliver the baby) | 1. Midwife 2. Nurse 3. Medical doctor 4. Medical Intern 5. Student 6. Health officer 7. Integrated emergency surgery officer 8. I do not know | Please check from the chart to confirm |
|  | What is the sex of the main birth attendant? | 1. Female 2. Male |  |
|  | At which time did you give birth? | 1. Daytime 2. Night time |  |

**Part IV Respectful maternity care related questions**

|  | **Physical abuse** | | |
| --- | --- | --- | --- |
| **SN** | **Question** | **Response** | **Remarks** |
|  | Did the birth attendants/the care providers use physical forces (**slapping, pinching, beating /hitting**) against you while you were in a labor pain? | 1. Yes 2. No | If yes, circle all applicable from the bolded |
|  | Did the birth attendant(s) threaten you with beating to let you obey their order? | 1. Yes 2. No |  |
|  | Have you tied down on a delivery bed when you were in labor? | 1. Yes 2. No |  |
|  | Did the health care provider(s) suture your perineum? | 1. No 2. Yes | If no, go to Q # 6 |
|  | If so, did they use local anesthesia so that it was pain-free? | 1. Yes 2. No |  |
|  | What birthing position do you prefer to give birth? | 1. Kneeling 2. Squatting 3. Lithotomy 4. Other ___ |  |
|  | Did the care providers allow you to assume the position of your choice during the current childbirth? | 1. No 2. Yes |  |
|  | Did the birth attendants(s) allow you to move around  (Ambulate) during the course of the labor? | 1. No 2. Yes | If yes, go to Q # 10 |
|  | If No, have they told you that you have a medical condition or you are in advanced labor or any other reason why they have not allowed you to do so? | 1. No 2. Yes |  |
|  | Did the birth attendants push your tummy down to deliver the baby (used fundal pressure)? | 1. Yes 2. No |  |
|  | Were you restricted from drinking any fluid throughout the labor course? | 1. Yes 2. No |  |
|  | Did the care providers order your caretakers/family to clean the delivery bed/room? | 1. Yes 2. No |  |

|  | **Non-consented Care** | | |
| --- | --- | --- | --- |
| **SN** | **Question** | **Response** | **Remarks** |
|  | Did the care provider introduce him/herself to you and your companion | 1. No 2. Yes |  |
|  | Did the care providers share the findings of your initial assessment with you and or your families? | 1. No 2. Yes |  |
|  | Did the care providers encourage you to ask questions? | 1. No 2. Yes |  |
|  | Did the care providers(s) explain to you what is being done and what to expect throughout the labor and birth process? | 1. No 2. Yes |  |
|  | Have you undergone an episiotomy? | 1. Yes 2. No | If No, go to Q # 7 |
|  | If Yes, did the birth attendant explain the indication and asked your permission/consent before she/he cut? | 1. No 2. Yes |  |
|  | Have you undergone a cesarean section? | 1. Yes 2. No | If No, go to Q # 9 |
|  | If Yes, did the care providers explain the indication and asked you to sign consent/ permission? | 1. No 2. Yes |  |
|  | Was your labor augmented? | 1. Yes 2. No 3. I don’t know | If 2 or 3, go to Q # 11 |
|  | If Yes, did the care providers explain the indication and asked your permission before putting you on the medication/oxytocin? | 1. No 2. Yes |  |
|  | Did you receive blood during the course of labor and delivery? | 1. Yes 2. No | If No, go to Q # 13 |
|  | If you were given blood, were you informed about the indication and was your/your families/ permission asked before the procedure is started | 1. No 2. Yes |  |
|  | Did the care providers coerce you to undergo C/S? | 1. Yes 2. No |  |

|  | **Non-confidential care** | | |
| --- | --- | --- | --- |
| **SN** | **Question** | **Response** | **Remarks** |
|  | Did the health care providers use curtains or other physical barriers so that your privacy was kept during the labor and delivery processes? | 1. No 2. Yes |  |
|  | Were other persons apart from the care providers allowed to the room you were giving birth who could observe you while you are naked on the bed? | 1. Yes 2. No |  |
|  | Did the birth attendants share your secret information with other non-concerned persons? Or don’t you trust them that your secret is likely to be shared with others? | 1. Yes 2. No 3. I don’t know |  |

|  | **Non-dignified Care** | | |
| --- | --- | --- | --- |
| **SN** | **Question** | **Response** | **Remarks** |
|  | Did the care provider speak to you politely throughout the course of the labor | 1. No 2. Yes |  |
|  | Did the care provider intimidate/ humiliate you at least one times? | 1. Yes 2. No |  |
|  | Did the care provider balm you for getting pregnant or shouting/crying due to the pain of the labor? | 1. Yes 2. No |  |
|  | Did the care provider shout at you to calm you down? | 1. Yes 2. No |  |
|  | Did the care providers allow your companion to enter the delivery room? | 1. No 2. Yes |  |

|  | **Discrimination based on specific patient attributes** | | |
| --- | --- | --- | --- |
| **SN** | **Question (Perceived discrimination)** | **Response** | **Remarks** |
|  | Did the care provider discriminate you because of your traditional belief? | 1. Yes 2. No 3. NA |  |
|  | Did the care provider discriminate you because of your religion? | 1. Yes 2. No |  |
|  | Did the care provider discriminate you because of your educational status? | 1. Yes 2. No 3. NA |  |
|  | Did the care provider discriminate you because you are from rural area/ from a very far distance? | 1. Yes 2. No 3. NA |  |
|  | Did the care provider discriminate you because you are RVI patient? | 1. Yes 2. No 3. NA |  |
|  | Did the care providers discriminate you because of your age? | 1. Yes 2. No |  |

|  | **Abandonment of Care** | | |
| --- | --- | --- | --- |
| **SN** | **Question** | **Response** | **Remarks** |
|  | Have you ever left alone without the care provider nearby you while you were in labor and needed help? | 1. Yes 2. No |  |
|  | Did you give birth in the health institution by yourself because the care providers were not around you? | 1. Yes 2. No |  |
|  | Have you encountered a life-threatening condition for which you have shouted for help but could not get anyone reached you in time? | 1. Yes 2. No |  |

|  | **Detention in Health facilities** | | |
| --- | --- | --- | --- |
| **SN** | **Question** | **Response** | **Remarks** |
|  | Did the health care providers detain you in the health facility because of payment of because you have pose damage to the property of the health institution? | 1. Yes 2. No |  |

Thank you very much for your cooperation!
