# Supplementary material for: Disrespect and abuse of women during childbirth in public health facilities in Arba Minch town, south Ethiopia – a cross-sectional study
Source: PLoS One. 2019 Apr 29;14(4):e0205545. doi: 10.1371/journal.pone.0205545 (PMC6488058; doi:10.1371/journal.pone.0205545)
Supplement: S3 File — (DOCX) [file pone.0205545.s003.docx]

## Operational definitions

**Physical abuse**: The presence of at least one of the following activities by the care provider on the client: beating, threatening with beating, slapping, pinching, restraining or tying down during labor, cutting or sutur­ing of episiotomy cuts or perineal tears without the use of anesthesia and the use of fundal pressure to fasten the delivery of the baby.

**Non-consented care:**  The presence of at least one of the following: providers not giving women or her relatives proper information about medical procedures, not asking for women’s permission to conduct medical procedures such as cesarean sections, episiotomies, hysterectomies, blood transfusions, tubal ligation, augmentation of labor; and coercing into a medical procedures such as a cesarean section.

**Non-confidential care**: The presence of at least one of the following: giving birth in a public view without privacy barriers such as curtains; and having healthcare providers share sensitive clients’ information, such as HIV status, age, marital status, and medical history, in a way that other people who are not involved in their care can hear.

**Non-dignified care:** A report by the client about at least one of the following: intentional humiliation, blaming, rough treatment, scolding, shouting at, women not allowed to bring a companion to the labor ward, and ordering to stop crying while they are in labor pain.

**Discrimination:** Discrimination based on specific client attributes like race, age, HIV/AIDS status, traditional beliefs and preferences, economic status, or educational status.

**Abandonment of care**: If there is any of the following practices: leaving laboring woman alone, women giving birth by themselves at health facilities, failure of caregivers to monitor women in labor and intervene in life-threatening conditions.

**Detention in facilities:** Detain the clients because of bills or damage to the property of the health care facility

**Grand multipara:** A woman who has given birth 5 or more times.

**Night time deliveries:** Deliveries that have occurred between 6:00 PM & 6:00 AM.
